# Evidence from 2016 Ethiopian demographic and health survey data: association between post health education maternal knowledge and neonatal danger signs

**DOI:** 10.1186/s12884-021-03681-0

**Published:** 2021-03-09

**Authors:** Mesfin Wudu Kassaw, Ayele Mamo Abebe, Biruk Beletew Abate, Seteamlak Adane Masresha, Ayelign Mengesha Kassie, Molalign Aligaz Adisu

**Affiliations:** 1 Department of nursing, college of health science, Woldia University, P.O Box 400, Woldia, Ethiopia; 2 Department of nursing, college of health science, Debre Berhan University, Debre Berhan, Ethiopia; 3 Department of public health, college of health science, Woldia University, Woldia, Ethiopia

**Keywords:** Maternal knowledge, Neonatal danger signs, EDHS, Association

## Abstract

**Background:**

Globally, 4 million infants die in their first 4weeks of life every year; above 8 million infants died before their first year of birthday, and nearly 10 million children died before their 5th birthday. Majority of the deaths were occurred at home because of not receiving health care. In Ethiopia, 120,000 infants died during their first 4 weeks of life. The aim of this study was to assess maternal knowledge about neonatal danger signs and its associations after they had been thought by health professionals in Ethiopia.

**Methods:**

This study used the 2016 Ethiopian Demographic and Health Survey data (EDHS) as a data source. The 2016 EDHS data were collected using a two stage sampling method. All the regions were stratified into urban and rural areas. The study sample taken from the 2016 EDHS data and used in this further analysis was 325. A logistic regression model was used to assess the associations with post health education maternal knowledge on neonatal danger signs.

**Results:**

In this study, mothers who had poor knowledge about neonatal danger signs (NDS) were 69.8 % (227) (95 %CI (64.8, 74.8 %). In the final logistic model, wanted no more child ((AOR = 4.15), (95 %CI = 1.12, 15.41)), female child ((AOR = 0.58), (95 %CI = 0.34, 0.98)), primary level maternal education ((AOR = 0.42), (95 %CI = 0.19, 0.92)), secondary level maternal education ((AOR = 0.37), (95 %CI = 0.16, 0.91)), and average size of child ((AOR = 2.64), (95 %CI = 1.26, 5.53)), and small size child ((AOR = 4.53), (95 %CI = 1.52, 13.51)) associated with post health education maternal knowledge about NDS.

**Conclusion:**

The mothers’ knowledge about NDS is poor even they were gave a birth in health facilities. Wanting of additional child, child sex, maternal education and size of child were associated with NDS knowledge. This indicates that the mode of health education provided for mother might not be appropriate and needs protocol changes.

## Background

Globally, 4 million babies die in their first 4weeks of life every year, and an equivalent number of babies were still-born (1). Nearly 130 million babies born every year but over 8 million infants died before their first birthday and 10 million infants died before their fifth birthday worldwide. Unfortunately, of these deaths, 98 % of deceases take placed in developing countries (2). Even it was late, the global neonatal mortality rate was declined from 36 deaths per 1000live-births in 1990 to 19 deaths per 1000live-births in 2015, and the number of neonatal deaths decreased from 5.1 million to 2.7 million, which was a remarkable decline in under-five child mortality since 2000, in which it saved the lives of 48 million under-five children (3). The highest numbers of neonatal deaths were in Sub-Saharan Africa (4). Majority of these neonatal deaths occurred at home and most of sick children were not taken to health facilities when they were sick that indicated only few families recognized signs of newborn illness or danger signs(5). In Ethiopia, 120,000babies died at their first 4weeks of life (6), and this neonatal mortality rate accounted for 42 % of under-five child deaths in the country, Ethiopia. However, the causes of neonatal mortality were not well documented in the country, Ethiopia. Yet, some studies reported sepsis, asphyxia, birth injury, tetanus, preterm birth and, congenital malformations as a causes of neonatal mortality (6, 7). Early screening of neonatal illness by identifying neonatal danger signs is an important step to improve survival of newborns (8). Evidences showed that the repeatedly reported neonatal danger signs were unable to breastfeed, movement on stimulation only, low or high temperature, tachypnea, severe chest in-drawing and history of convulsion(9). Newborns that experienced danger signs were 2times more likely to die compared to those never experienced any danger signs (10). A study from Indonesia also reported that neonatal illness during the first month of life, and poor maternal knowledge about neonatal danger signs were risk factors for neonatal mortality (11). For the identification of NDS, different tools were introduced into the health programs in several countries including Ethiopia. Integrated Management of Newborn and childhood illness (IMNCI) was the one that focused on assessment of neonatal danger signs and applies prompt and timely action (9, 12). As per the IMNCI recommendation, reducing neonatal morbidity and mortality requires caregiver’s recognition of suggestive neonatal danger signs and visiting a clinic for early management (9, 12). In Ethiopia, mothers are the primary caretakers for the majority of neonates (9, 13). This means, maternal knowledge concerning neonatal danger sign is very important in saving neonates, and reducing child morbidity and mortality (9). However, evidences across the world including Ethiopia indicated that mothers’ knowledge on neonatal danger signs was still low (9, 14–16). Unfortunately, low level of knowledge about neonatal danger signs mentioned as a major barrier to seek early treatment (17) that eventually leads to neonatal mortality (10, 18). Although, various studies had examined magnitude, determinants of neonatal mortality, and NDS (17, 19), there are no studies on post health education maternal knowledge about neonatal danger signs (1, 4, 6). The aim of this study was to assess post health education maternal knowledge and association on neonatal danger signs among mothers who gave birth in health facilities.

## Methods

### Study design, and sampling procedure

The 2016 Ethiopia Demographic and Health Survey data was the source for this study. The 2016 EDHS data used a two-stage stratified sampling design. The first stage contained 645 enumeration areas that derived from 2007 Ethiopia population and housing census (PHC) database. A total of 18,008 households were considered, and of which 16,650 (98 % of response rate) households were eligible. The details about the study population were stated by Wado with a description how women involved (20). The data had a nationally representative sample of women (15–49 years), children (birth- 5 years), and men (15–59 years) from the nine administrative regions and two administrative cities of Ethiopia (20).

### Data collection tools, procedures and population

The data collection period for this survey was from 18 January to 27 June, 2016. The 2016 EDHS data used 5groups of questionnaires: These questionnaires are the household questionnaire, the woman’s questionnaire, the man’s questionnaire, the biomarker questionnaire, and the health facility questionnaire (21). The questions about children were integrated into woman’s questionnaires. In this study, 325 mother-child pairs were included after managing the EDHS data.

#### Pretest

The 2016 EDHS conducted a pretest from October 1–28, 2015, in Bishoftu at the Asham African Training Centre. The field practice was conducted on clusters surrounding Bishoftu that were not included in the 2016 EDHS sample. Following the field practice, a debriefing session was held with the pretest field staffs, and modifications to the questionnaires were made based on lessons drawn from the pretest.

#### Training and field works

 Team supervisors, field editors, interviewers, secondary editors, and reserve interviewers were recruited by central statistical agency (CSA). The training consisted of instructions regarding interviewing techniques and field procedures, a detailed review of questionnaire content, and electronic questionnaires. In addition, other teams were recruited and trained to collect biomarker data, including taking height and weight measurements, testing for Anemia, and preparing dried blood spots for a laboratory. The selection of team supervisors and field editors was based on their experience and their performance during the pretest and the main training session.

### Variables

The outcome variable of this study is post health education maternal knowledge about NDS. The independent variables include socio-demographic characteristics of children and mothers, health care services, and substance use. However, this study considers only the variables that are available in the 2016 EDHS data.

#### Measurement of variables

The 2016 EDHS data had 8 dichotomize questionnaires that addressed the post health education maternal knowledge about NDS. Those 8 questionnaires were adopted from the UNICEF and WHO’s NDS questionnaires (22). The 8 NDS questionnaires are S440BA, S440BB, S440BC, S440BD, S440BE, S440BF, S440BG, and S440BH. These 8 questionnaires were used to collect a data from mothers using **yes** and **no** options.

#### Definition

##### Neonatal danger signs (NDS)

Complain of mothers that their child had either or all of the 8 neonatal danger signs.

##### Knowledge about NDS

Assessment of what mothers described about NDS using the aforementioned 8 questions.

##### Good knowledge about NDS

Mothers who scored above the median value for the knowledge questions.

##### Poor knowledge about NDS

Mothers who scored below the median value for the knowledge questions.

### Data analysis

The data were extracted, edited, and analyzed using SPSS version 23. A weighted analysis was conducted using the same sampling weight given for each region in the Ethiopia DHS data to compensate for the unequal probability of selection between the strata (21). Socio-demographic characteristics of study participants were summarized by descriptive statistics. Bivariate logistic regression was performed and variables with a p-value of less than 0.25 were transported to multivariable logistic regression model to identify factors associated with maternal knowledge about neonatal danger signs. Variables with *p*-values of < 0.05 in the multivariable logistic regression model were considered as statistically significant.

## Results

### Socio‐demographic status

In this study, all the mothers have no history of smoking. Regarding occupation 167(51.4 %) mothers did not work at all (they spent in home) as housewife. From 325 mothers 268(82.5 %) households headed by males. Almost all 322(99.1 %) mothers were married, 156(48.0 %) mothers were Orthodox follower, and 191 (58.8 %) were from a richest households (Table 1).

**Table 1:** The socio-demographic status of respondents and their under-five years old children in Ethiopia

Variables	Categories	Frequency (*n*=325)	Percent
Husband occupation	Did not work	14	4.3
	Professional/managerial	55	16.9
	Clerical	3	0.9
	Sales	59	18.2
	Agricultural employee	96	29.5
	Service	26	8.0
	Skilled manual	56	17.2
	Unskilled manual	16	4.9
Maternal occupation	Did not work	167	51.4
	Professional/managerial	24	7.4
	Clerical	9	2.8
	Sales	52	16.0
	Agricultural employee	42	12.9
	Service	12	3.7
	Skilled manual	14	4.3
	Unskilled manual	5	1.5
Head of household	Male	268	82.5
	Female	57	17.5
Residence	Urban	172	52.9
	Rural	153	47.1
Maternal age	15-19	11	3.4
	20-24	69	21.2
	25-29	96	29.5
	30-34	81	24.9
	35-39	42	12.9
	40-44	17	5.2
	45-49	9	2.8
Maternal education	No education	92	28.3
	Primary	124	38.2
	Secondary	60	18.5
	Higher	49	15.1
Region	Tigray	71	21.8
	Afar	2	0.6
	Amhara	10	3.1
	Oromia	14	4.3
	Somali	6	1.8
	Benishangul-Gumz	30	9.2
	SNNPR	43	13.2
	Gambela	5	1.5
	Harari	54	16.6
	Addis-Adaba	61	18.8
	Dire-Dawa	29	8.9
	Orthodox	156	48.0
	Catholic	2	0.6
	Protestant	47	14.5
	Muslim	119	36.6
	Traditional	1	0.3
	Other	156	48.0
Wealth index	Poorest	15	4.6
	Poorer	31	9.5
	Middle	38	11.7
	Richer	50	15.4
	Richest	191	58.8
Readiness	Wanted then	273	84.0
	Wanted later	38	11.7
	Wanted no more	14	4.3
Chat chewing	No	289	88.9
	Yes	36	11.1
Type of birth	Single birth	316	97.2
	1^st^ of multiple	9	2.8
Sex of the child	Male	163	50.2
	Female	162	49.8
Marital status	Married	322	99.1
	Living with partner	3	0.9
Fast and rapid breathing	No	294	90.5
	Yes	31	9.5
Size of the child at birth	Very large	71	21.8
	Large	50	15.4
	Average	153	47.1
	Small	27	8.3
	Very small	24	7.4
KMC (Kangaroo mother care)	No	78	24.0
	Yes	247	76.0

Note: SNNPR-Southern nations, nationalities and peoples representatives

### Prevalence of neonatal danger signs (NDS)

In this study, mothers who had poor knowledge about NDS after they received health education was 227(69.8 %), 95 %CI (64.8, 74.8 %).

### Association of post health education maternal knowledge about NDS in Ethiopia

Wealth index, size of child, maternal education, child sex, and residence were associated with post health education maternal knowledge at bivariate logistic regression model. However, in the final multivariate logistic regression analysis, wontedness of child ((AOR), (95 %CI), wanted no more((4.15), (1.12, 15.41) child sex ((AOR), (95 %CI), female((0.58), (0.34, 0.98), maternal education((AOR), (95 %CI), primary ((0.42), (0.19, 0.92), secondary ((0.37), (0.16, 0.91), and size of child((AOR), (95 %CI), average ((2.64), (1.26, 5.53), and small ((4.53), (1.52, 13.51) associated with post health education maternal knowledge about NDS (Table 2).

**Table 2:** The association of post health education maternal knowledge about neonatal danger signs in Ethiopia

Variable	Categories	Neonatal Danger Signs Awareness (*n*=325)				95% CI		*P*-value
		Poor	Good	COR	AOR	Lower	Upper	
Residence	Rural	108 (62.8)	64 (37.2)	0.48*	0.53	0.28	1.02	0.06
	Urban	119 (77.8)	34 (22.2)	1	1	-	-	1
Head of household	Male	189 (70.5)	79 (29.5)	1	1	-	-	1
	Female	38 (66.7)	19 (33.3)	1.20	1.31	0.66	2.60	0.44
Was child wanted	Wanted then	196 (71.8)	77 (28.2)	1	1	-	-	1
	Wanted latter	23 (60.5)	15 (39.6)	1.66	1.58	0.71	3.49	0.26
	Wanted no more	8 (57.1)	6 (42.9)	1.91	4.15	1.12	15.41	0.03
Khat Chewing	No	203 (70.2)	86 (29.8)	1	1	-	-	1
	Yes	24 (66.7)	12 (33.3)	1.18	1.31	0.56	3.06	0.54
Child sex	Male	105 (64.4)	58 (35.6)	1	1	-	-	1
	Female	122 (75.3)	40 (24.7)	0.59*	0.58	0.34	0.98	0.04
Child birth	Single birth	220 (69.6)	96 (30.4)	1	1	-	-	1
	2^nd^ of multiple	7 (77.8)	2 (22.2)	0.66	0.59	0.11	3.26	0.54
Maternal education	No education	72 (78.3)	20 (21.7)	0.27*	0.43	0.17	1.14	0.09
	Primary	88 (71.0)	36 (29.0)	0.39*	0.42	0.19	0.92	0.03
	Secondary	43 (71.7)	17 (28.3)	0.38*	0.37	0.16	0.91	0.03
	Higher	24 (49.0)	25 (51.0)	1	1	-	-	1
Caesarean Section	No	199 (71.6)	79 (28.4)	1	1	-	-	1
	Yes	28 (59.6)	19 (40.4)	1.71	1.31	0.61	2.81	0.48
Size of child	Very large	55 (77.5)	16 (22.5)	1	-	-	-	1
	Large	36 (72.0)	14 (28.0)	1.34	1.93	0.76	4.91	0.17
	Average	104 (68.0)	49 (32.0)	1.62	2.64	1.26	5.53	0.01
	Small	14 (51.9)	13 (48.1)	3.19*	4.53	1.52	13.51	0.005
	Very small	18 (75.0)	6 (25.0)	1.15	1.46	0.44	4.85	0.54
ARDS	No	207 (70.4)	87 (29.6)	1	1	-	-	1
	Yes	20 (64.5)	11 (35.5)	1.31	1.06	0.43	2.63	0.90
KMC	No	50 (64.1)	28 (35.9)	1	1	-	-	1
	Yes	177 (71.7)	70 (28.3)	0.71	0.67	0.36	1.26	0.22
Maternal age	15-19	7 (63.6)	4 (36.4)	1.14	1.53	0.18	12.97	0.70
	20-24	40 (58.0)	29 (42.0)	1.45	1.83	0.32	10.37	0.50
	25-29	68 (70.8)	28 (29.2)	0.82	0.79	0.15	4.30	0.78
	30-34	60 (74.1)	21 (25.9)	0.70	0.70	0.13	3.84	0.68
	35-39	34 (81.0)	8 (19.0)	0.47	0.39	0.06	2.40	0.31
	40-44	12 (70.6)	5 (29.4)	0.83	0.46	0.06	3.51	0.46
	45-49	6 (66.7)	3 (33.3)	1	-	-	-	1

Note: *=*p*<0.05, *ARDS* Acute respiratory distress syndrome, *KMC* Kangaroo mother care

## Discussions

The aim of this study was to assess the prevalence and association of post health education maternal knowledge about neonatal danger signs in Ethiopia. In this study, the post health education maternal poor knowledge about NDS was 227(69.8 %), 95 %CI (64.8, 74.8 %) and good knowledge was 98(30.2 %), 95 %CI (25.4, 35.6). The present study’s post health education maternal good knowledge about NDS was higher than a study reported from Woldia general hospital that reported 11.67 % of good knowledge about NDS (22). Similarly, the study from Woldia general hospital reported the prevalence of poor knowledge about NDS, 88.3 % that indicated a high level than the present study (22). The difference might be as a result of study population variation. We consider only mothers who gave birth in health facilities under the assistance of trained health professionals, but the referred paper’s study population were mothers who gave birth in any settings (health facilities, home or elsewhere)(22). The current study has higher prevalence of good knowledge about NDS than a study conducted in Northwest Ethiopia, 18.2 % (9). This difference might also as a result of study setup variation. The study from northwest Ethiopia was conducted in a community, but the current one studied in health facilities only. The present study’s prevalence of poor knowledge is consistent with a study conducted in Wardha, India, 67.4 % (23), even the study from India considered both community and health facility settings. The similarity might be because of socio-demographic variation. The Indian communities have better socio-demographic status that might decrease the poor level of knowledge about NDS in Indian even the study consider both health facilities, and communities. But the Ethiopian mothers’ level of good knowledge about NDS after they receive health education about it is still low that display the low socio-economic status of the country. The current study’s prevalence of good knowledge is lower than studies conducted in Arba-Minch Hospital, 40.9 % (24), and Chencha district, 50.3 %(25). The difference might be as a result of inclusion and exclusion criteria. In the current study mothers who had not gave birth under a trained health professionals were excluded but in the former studies, they were not excluded. The current study’s prevalence of good knowledge about NDS was in-lined with a study that reported 31.32 % (26) from Wolkitie, and 32.4 % from Gedeo zone (27), and other study that considered four regions of Ethiopia (15). The similarity between these studies might be as a result of study population variation. All the studies including our study consider mothers from both rural and urban areas. The current study’s prevalence of good knowledge about NDS was in lined with a studies conducted in Afghanistan (28), and Nigeria, 30.3 % (29). However, the studies from Afghanistan and Nigeria considered both community and health facilities. The similarity might be because of socio-demographic difference; even the difference between those countries, particularly in economy might not be too much. The socio-demographic difference might exhibited by better awareness about NDS in both mothers who visit health facilities and stay at home within the communities than Ethiopia. Mothers who did not wanted the birth ((AOR), (95 %CI), ((4.2), (1.12, 15.4) than mothers who wanted the birth were had poor knowledge about NDS. This study’s degree of association was supported by a study from India that indicated mothers who had not prepared and planed for birth were more likely to have poor knowledge about NDS (30). This might be because of that mothers’ interest to learn, and give time, and concern for unplanned birth is low. In addition, in case of unplanned birth mothers might face challenges like workload, financial and care issues after birth. All these factors might affect mothers’ knowledge about NDS directly or indirectly. Mothers who complete primary ((AOR), (95 %CI), ((0.42), (0.19, 0.92), and secondary ((AOR), (95 %CI), ((0.38), (0.16, 0.91) education were had good knowledge about NDS than mothers who complete higher education. This degree of association contradicts with other studies that reported a lack of association between maternal knowledge regarding NDS, and maternal education (22). This might be because of that mothers who had higher education might have jobs out of home than primary and secondary drop-out mothers. This might decrease the opportunities of mothers with higher education to know about NDS, in which their children spent the day with care givers. Mothers who had female children ((AOR), (95 %CI), ((0.58), (0.34, 0.98) compared to male children had good knowledge about NDS. Although, we did not get evidence on this degree of association, the reason might be because of sample size limitation. While we prepare the EDHS data, the sample size decreased significantly that might cause a false association. Mothers who had average child size ((AOR), (95 %CI), ((2.64), (1.26, 5.53) and small child size ((AOR), (95 %CI), ((4.53), (1.52, 13.51) were had poor knowledge about NDS than mothers who gave very large size. Just like the association of NDS and sex, we did not get evidence on the association of child size and NDS. However, the justification might be that very large babies could have co-morbidities and required the concern of mothers to seek medical care. This frequent maternal follow up might improve their knowledge about NDS.

## Conclusions

 The mothers’ knowledge about NDS is unsatisfactory even they were gave a birth in a health facility. This indicates that the mode of health education provided for mother might not be appropriate and needs protocol changes. Wontedness of child, child sex, maternal education and size of child associated with NDS knowledge. The government should assess the quality of health education and the need for protocol change about neonatal danger signs.

### Limitation

The limitation of this study is missing of pertinent variables while we clean the data. Due to this, the association of NDS knowledge and possible predictors might not be checked. However, this is a nation based evidence on maternal knowledge after they had been learnt about it during ANC, and postpartum, which will be used for program design and other interventions.

## Data Availability

Most of the data generated or analyzed during this study are included in this published article. However, the original datasets generated and/or analyzed during the current study are available in the DHS data set repository, .

## Abbreviations

S440BA: Which danger sign of newborn health were you told about: feeding less?
S440BB: Which danger sign of newborn health were you told about: too cold or too hot?
S440BC: Which danger sign of newborn health were you told about: too sleepy?
S440BD: Which danger sign of newborn health were you told about: convulsion?
S440BE: Which danger sign of newborn health were you told about: fast breathing?
S440BF: Which danger sign of newborn health were you told about: umbilicus red?
S440BG: Which danger sign of newborn health were you told about: pus in eye?
S440BH: Which danger sign of newborn health were you told about; fever
AOR: Adjusted odds ratio
CI: Confidence interval
SPSS: Statistical package for social science
EDHS: Ethiopian Demographic and Health Survey data
EA: Enumeration areas
SNNPR: Southern nations, nationalities, and people of Ethiopia
